# Human monkeypox expansion from the endemic to non-endemic regions: Control measures

**DOI:** 10.1016/j.amsu.2022.104048

**Published:** 2022-06-23

**Authors:** Hashaam Jamil, Waleed Tariq, Muhammad Junaid Tahir, Rabia Salman Mahfooz, Muhammad Sohaib Asghar, Ali Ahmed

**Affiliations:** a Lahore General Hospital, Lahore, 54000, Pakistan; b Shalamar Institute of Health Sciences, Lahore, Pakistan; c Dow University of Health Sciences–Ojha Campus, Karachi, Pakistan; d School of Pharmacy, Monash University, Jalan Lagoon Selatan, Bandar Sunway, 47500, Subang Jaya, Selangor, Malaysia

**Keywords:** Zoonosis, Infection, Vaccination, Virology, Epidemic, Public health

## Abstract

The recent monkeypox outbreak in non-endemic countries is a rare event. It is a zoonosis with clinical features similar to smallpox, transmitting from animal to human and human to human. Since 2000, the number of monkeypox cases in humans had increased in African countries, resulting in its spread to the other parts of the world. On May 26, 2022 World Health Organization has confirmed 120 cases of monkeypox in 23 non-endemic countries. The decreased immunity to the *Orthopoxviruses*, human migration from endemic to non-endemic regions, genetic mutations in the viral genome, and reduced surveillance may contribute to the recent outbreaks. A multi-pronged approach comprising health education, tracking human migration, developing diagnostic facilities, and an effective vaccine could prevent transmission and pathogenicity.

The recent monkeypox outbreak in non-endemic regions is a rare event. Monkeypox is a zoonosis caused by the monkeypox virus (MPXV), a member of the *Orthopoxvirus* genus. The MPXV resembles to the Variola virus, the cause of smallpox. MPXV was first identified as a human pathogen in the Democratic Republic of the Congo (DRC) in 1970 [1]. There are two genetic clades of MPXV that have been identified so far: Western African and Central African. The Central African clade has a greater case fatality rate (11%) as compared to the Western African clade (1%) and has more human-to-human transmission [2].

Human monkeypox has clinical characteristics that are remarkably similar to smallpox. Most individuals exhibit prodromal illness with fever, malaise, and swollen lymph nodes after an incubation period of 10–14 days. Over the course of next 14–21 days, the normal human monkeypox rash begins as maculopapular lesions and progresses through papular, vesicular, pustular, and crust phases before sloughing and leaving dyspigmented scars [3]. Complications such as subsequent skin infections, bronchopneumonia, sepsis, encephalitis, and corneal infection leading to blindness are possible [4]. Animal–human transmission and human–human transmission are the two possible modes of MPXV transmission. Inter-human transmission has been linked via respiratory droplets, contact with bodily fluids, a contaminated patient's environment or belongings, an infected person's skin lesion and with sexual transmission from an infected individual with groin and genital lesions. Animal-human transmission occurs via direct contact with natural viral hosts or consumption of hosts. Additionally, zoonotic transmission can occur by direct contact with an infected animal's blood, bodily fluids, or inoculation through mucocutaneous lesions [5]. Diagnosis is mainly clinical, but laboratory diagnosis such as viral isolation, culture, immunohistochemistry for viral antigen detection, ELISA for antibodies detection (IgG and IgM), and specific viral DNA detection using PCR are required for the definitive diagnosis [6]. Treatment is mainly symptomatic. Currently, no licensed antiviral drug is available for MPXV infection. Vaccination combined with an aggressive surveillance program can help in the eradication of MPXV [3].

There are mounting concerns about the geographical spread and further resurgence of Monkeypox. Monkeypox outbreaks have been reported in 11 African countries in recent years, with the DRC reporting the most cases. Beginning in 2000, the DRC began reporting several suspected cases, which climbed from >10,000 cases in 2000–2009 to >18,000 cases in 2010–2019, with an additional 4594 suspected cases recorded in the first nine months of 2020 alone. In Nigeria, the number of cases had drastically increased from three in 1970's to 181 in 2017–2019 [7]. In 2003, the United States reported the sole human case of monkeypox outside of Africa, that was associated with rodents imported from Ghana. The first human-human transmission occurred in September 2018, when 3 travelers infected with MPXV left Nigeria and arrived in two countries, two cases in UK and one case in Israel [8]. In 2019, a Nigerian national became infected with MPXV in Singapore. Few cases were also transported in the UK and US during 2021. Multiple cases of monkeypox were discovered in various non-endemic countries during the recent outbreak, which began in May 2022. According to World Health Organization (WHO), on 21^st^ May 2022, 92 laboratory confirmed cases, and 28 suspected cases have been reported to WHO from 12 countries that are non-endemic for MPXV [9]. After 5 days, on 26^th^ May 2022, a cumulative total of 257 laboratory confirmed cases and around 120 suspected cases have been reported to WHO from 23 countries that are non-endemic for MPXV [10].

The specific justification for these expanded cases in endemic and non-endemic regions isn't known. Multiple theories have been proposed to explain the rise in monkeypox cases. First, Smallpox immunization was discontinued in 1980, resulting in evasion of immunity to the Orthopoxviruses. Following vaccine discontinuation after smallpox eradication and declining immunity provided an immunologic niche for monkeypox. Second, since 1980, the DRC has experienced significant anthropogenic and demographic changes, which may have increased the local population's exposure to MPXV reservoir species. Furthermore, repeated wars, civil turmoil, and poverty cause the affected populace to escape and eventually seek refuge more profound in the rainforest, relying drasticalluy upon bush food, which also increases the transmission rate. Third, an expanded resurgence in humans, particularly in immunocompromised hosts, may allow MPXV to acquire genetic mutations that increment its fitness in human hosts, potentially leading to increased transmission,virulence and pathogenic potential. Fourth, surveillance for human monkeypox infections in endemic areas is quite challenging. Restricted assets, inadequate infrastructure, insufficient diagnostic tools, and lack of clinical facilities in recognizing monkeypox illness are some of the challenges encountered by the surveillance framework.

A multi-pronged approach can lessen the burden of human monkeypox infection in endemic and non-endemic regions. The health education campaign, seminars, pamphlets, and workshops would be useful in disseminating information, especially in endemic areas. There is a strict need to establish quarantine areas and isolation for the infected person. The wildlife trade should be banned, to control the migratory activities of animals from endemic areas. As international travel continues to increase, the travelling from endemic to non-endemic areas should be strictly monitored. Public health officials should assess potential hazards and track passengers or others who may have had contact with an infectious patient while in transit. Globally, there are few laboratories equipped with facilities to confirm monkeypox infection. Adequate diagnostic facilities must be provided to improve early detection. Vaccination is still a viable control option; however, reintroducing Vaccinia vaccination as part of routine immunization programs would be exceedingly difficult logistically.

Monkeypox is no longer regarded as a rare disease. Following its recent outbreak, it has become a worldwide health security threat, necessitating increased vigilance to prevent its spread. Appropriate interventions and active surveillance efforts are urgently needed to avoid transmission and pathogenicity. It is high time that national organizations, national health departments, health care providers, researchers, and the media sector collaborate with international organizations to establish and implement a strategic framework for monkeypox awareness and prevention.

## Ethical approval

Not required.

## Sources of funding

None.

## Authors’ contributions

M.J.T, H.J, and A.A conceived the idea, H.J, W.T, R.S.M, M.S.A, and M.J.T retrieved the data, did write up of letter, and finally, A.A, M.S.A, W.T, and M.J.T reviewed and provided inputs. All authors approved the final version of the manuscript.

## Registration of research studies

1. Name of the registry: Not required.
2. Unique Identifying number or registration ID: N/A
3. Hyperlink to your specific registration (must be publicly accessible and will be checked):

## Guarantor

Muhammad Sohaib Asghar.

## Consent

Not applicable.

## Financial disclosure

No financial support was provided relevant to his article.

## Provenance and peer review

Externally peer reviewed, not commissioned.

## Declaration of competing interest

None.
